# Unusual case of follicular lymphoma of vocal cord

**DOI:** 10.1007/s00428-026-04443-z

**Published:** 2026-02-23

**Authors:** Griffin H. Willman, Zohre Sadeghian, Jyotsna Kochiyil, Chieh Lin Fu, Samuel Trosman, Gulrukh Botiralieva, Natalya Pak, Yaroslav N. Chernov, Shukhrat A. Abdukhalikov, Amir Behdad, Kirill A. Lyapichev

**Affiliations:** 1 Charles E. Schmidt College of Medicine, Florida Atlantic University, Boca Raton, FL USA; 2 Department of Pathology and Laboratory Medicine, Cleveland Clinic Florida, 2950 Cleveland Clinic Boulevard, Weston, FL 33331 USA; 3 Department of Imaging, Cleveland Clinic Florida, Weston, FL USA; 4 Department of Hematology and Oncology, Cleveland Clinic Florida, Weston, FL USA; 5 Department of Head and Neck Surgery, Cleveland Clinic Florida, Weston, FL USA; 6 IPSUM Pathology LLC, Tashkent, Uzbekistan; 7 Central Asian University, Tashkent, Uzbekistan; 8 Medcloud, Tashkent, Uzbekistan; 9 The Institute for Urology and Reproductive Health, I. M. Sechenov First Moscow State Medical University (Sechenov University), Moscow, Russia; 10 Impuls Medical Institute Chirchiq Branch, Tashkent, Uzbekistan

**Keywords:** Classic follicular lymphoma, Vocal cord, Larynx

## Abstract

Primary laryngeal lymphoma is a rare diagnosis, accounting for less than 1% of all laryngeal tumors. Approximately 100 cases have been reported in the English-language literature. A variety of lymphoma classifications have been described, including diffuse large B-cell lymphoma, NK/T-cell lymphoma, Burkitt lymphoma, lymphoplasmacytic lymphoma, mucosa-associated lymphoid tissue (MALT) lymphoma, mantle cell lymphoma, marginal zone lymphoma, and peripheral T-cell lymphoma. To our knowledge, follicular lymphoma involving the larynx has been rarely reported. We report a case of a 74-year-old woman who presented with a 1-year history of hoarseness refractory to semi-occluded vocal tract (SOVT) therapy and was diagnosed with follicular lymphoma involving the false vocal fold. At presentation, the patient felt well and did not have any B symptoms. Initial computed tomography (CT) scan of the neck did not show any lesions, but a subsequent videolaryngostroboscopy revealed a small lesion in the false vocal folds. The lesion was surgically excised, and histological evaluation revealed features consistent with a diagnosis of follicular lymphoma. Herein, we present a rare case of primary follicular lymphoma of the larynx and describe its histological and immunophenotypic characteristics. Furthermore, we conducted an extensive review of the English-language literature and discuss comparable published cases.

## Introduction

Follicular lymphoma (FL) is one of the most common subtypes of non-Hodgkin lymphoma in the USA and Western Europe, accounting for approximately 20–30% of all non-Hodgkin lymphomas [1–4].

FL mainly occurs in adults, with a median age of about 65 years, and shows no gender predilection [5]. It is a tumor derived from B-cells in germinal centers and typically demonstrates a nodular appearance on gross specimens and microscopically. FLs are classically characterized by overexpression of BCL2, an oncoprotein with an inhibitory effect on cellular apoptosis. The majority of FLs show at least some follicular architectural pattern and consist of a mixture of centrocytes and centroblasts. Approximately 85% carry the t (14;18) (q32; q21) translocation. These tumors are classified as classic FL, distinguishing them from less common variants. The 5th edition of WHO recognizes subtypes including classic FL (cFL), FL with atypical cytologic features (uFL), FL with mainly diffuse growth (dFL), and follicular large B-cell lymphoma (FLBCL) [6]. Most patients present with disseminated disease at diagnosis, typically involving both peripheral and central (abdominal and thoracic) lymph nodes, as well as splenomegaly. Although peripheral lymphadenopathy is the most common presentation, any nodal region may be affected. Exclusively extranodal disease is rare but can arise in nearly any extranodal location [7–9].

Many patients are asymptomatic, and B symptoms such as fever and weight loss are uncommon. FL primarily involves lymph nodes, with frequent extension to the spleen and bone marrow. Less commonly, the Waldeyer ring and peripheral blood may be affected. Other sites that may be involved include the gastrointestinal tract, often accompanied by soft tissues, thoracic vertebrae, breast, and ocular adnexa [10]. Follicular lymphoma primarily arising in the larynx is extremely rare and has not been previously reported in the literature to our knowledge.

In this report, we present a case of follicular lymphoma originating in the larynx in a woman with a chief complaint of hoarseness. We also conduct a literature review through PubMed and analyze the implications of our case in the context of the broader literature.

## Case presentation

A 74-year-old woman with no pertinent past medical history presented to the otolaryngology department for evaluation of a 1-year history of dysphonia. The patient was previously seen by an outside institution otolaryngologist, who referred her to vocal therapy with a diagnosis of right muscle tension dysphonia with significant right false vocal fold hooding. The original computed tomography (CT) scan of the neck showed no abnormalities and the patient was referred to a speech language pathologist for 9 sessions including Semi-Occluded Vocal Tract (SOVT) therapy with no improvement. She denied any symptoms of dysphagia or pain with speaking and never smoked tobacco products. Physical examination revealed no masses or abnormalities, and no lymphadenopathy was present. The complete blood count (CBC) and basic metabolic panel (BMP) were within normal limits.

**Fig. 1:**
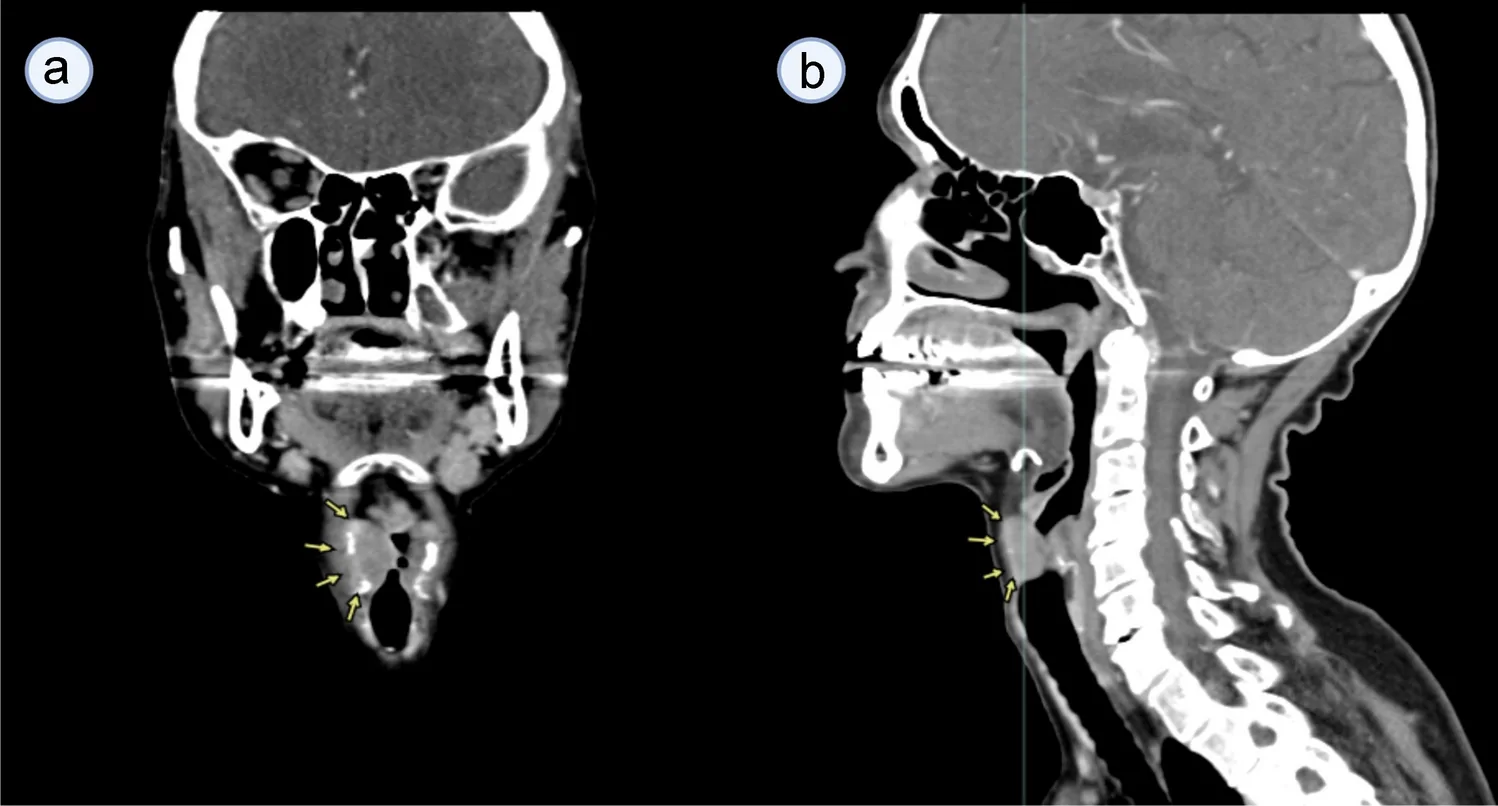
**a** Coronal contrast-enhanced CT image demonstrating extension of the isodense mass into the right paraglottic fat and thyroid cartilage (yellow arrows). **b** Sagittal contrast-enhanced CT image showing superior-to-inferior extension of the laryngeal mass from the level of the epiglottis to the subglottis

Consequent flexible videolaryngostroboscopy was performed, which revealed submucosal swelling of the right false vocal fold, initially thought to be suspicious for a saccular cyst or laryngocele. A new CT neck with contrast revealed a mass in the right laryngeal ventricle measuring 1.2 × 0.6 × 2.0 cm extending into the right paraglottic fat up to the ala of thyroid cartilage and laryngeal surface of the epiglottis into the petiole and inferiorly up to the glottis (Fig. 1).

Mass effect in the supraglottic region was noted with hyperdense tissue in the pre-epiglottic fat. Additionally, there was an interval increase in size of the right level 3 cervical lymph node measuring 1.9 × 1.2 cm. Several other nonspecific subcentimeter lymph nodes were noted new from previous scans. Direct microlaryngoscopy was recommended to the patient, and the lesion was biopsied.

**Fig. 2:**
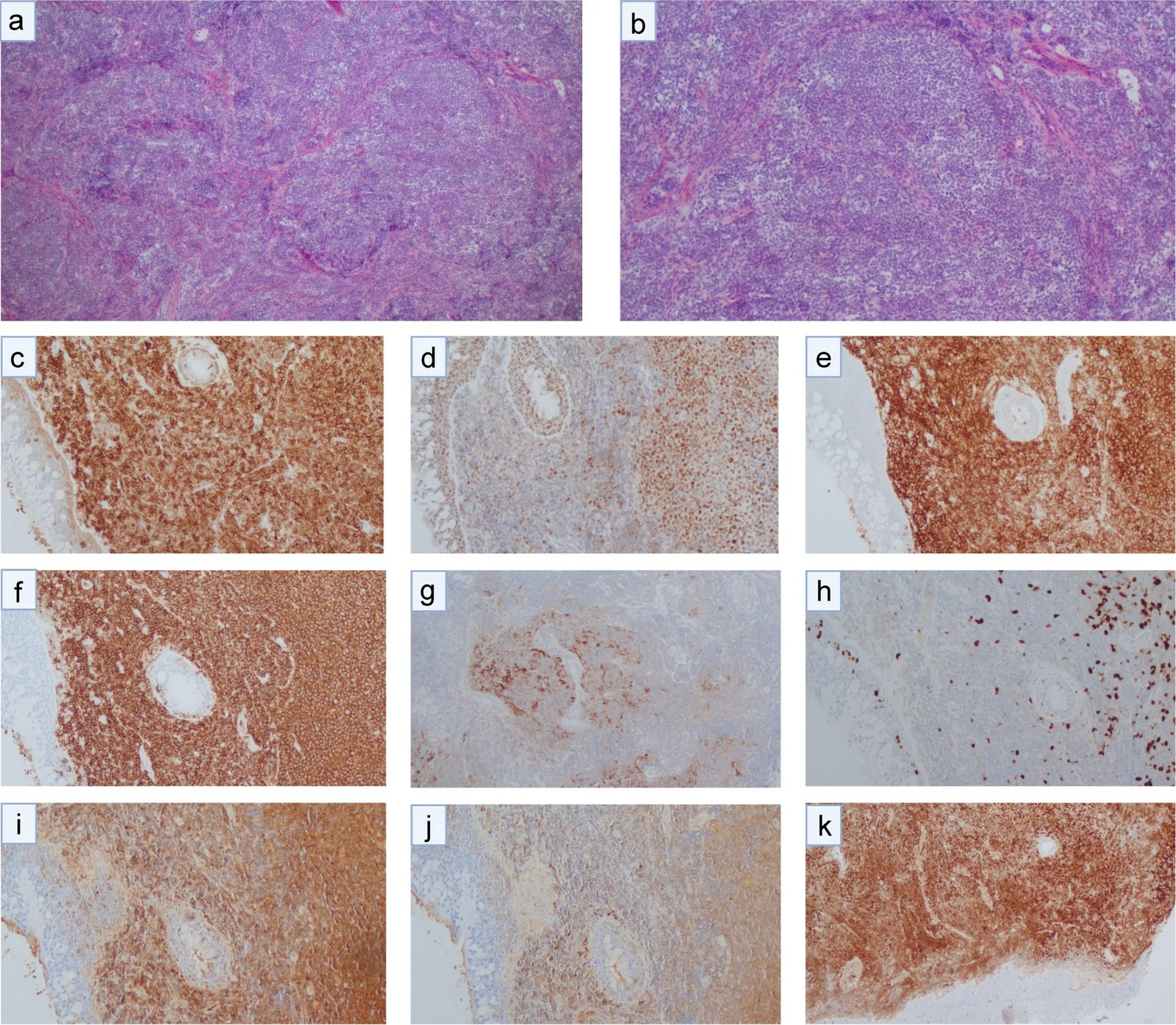
Histopathological and immunohistochemical (IHC) analysis of follicular lymphoma. **a** Low-power view (×10 magnification) of the biopsy with Hematoxylin and Eosin (H&E) staining, showing characteristic follicular architecture. **b** Higher magnification (×20) reveals details of lymphoid follicles with a mixture of mostly centrocytes and scattered centroblasts, consistent with low grade (1–2) follicular lymphoma. **c**–**k** IHC staining at ×20 magnification highlights specific markers: **c** BCL2, **d** BCL6, **e** CD10, **f** CD20, **g** CD21, **h** Ki-67, **i** kappa, **j** lambda, and **k** PAX5

Histological sections showed fragments of vocal cord mucosa effaced by atypical, follicular lymphoid proliferation (Fig. 2a, b). Immunohistochemical studies (IHC) were performed on unstained tissue sections obtained from the paraffin-embedded biopsy specimen. The neoplastic cells are positive for CD10, CD20, BCL2, BCL6, and PAX5 and negative for CD5 and CyclinD1 (Fig. 2c–f and k). CD3 and CD5 stain infiltrating small T-cells. CD21 highlights follicular dendritic cell meshwork within neoplastic follicles (Fig. 2g). The Ki-67 stain showed an overall proliferation rate of 20–30% (Fig. 2h). Kappa and Lambda light chains immunohistochemistry was performed and shows Kappa light chain restriction (Fig. 2i and j).

Flow cytometry analysis was unsuccessful due to an inadequate number of viable cells.

These findings are consistent with a diagnosis of classic FL. The otolaryngologist and primary care physician were notified, and an oncology consultation was suggested.

The consequent biopsy of metabolically active deep cervical nodes showed progression of the follicular lymphoma with no signs of transformation. Primary extranodal lymphoma staging (using the Lugano/Ann Arbor systems) distinguishes between localized disease (Stage 1E) and more extensive, yet still limited, spread (Stage 2E), both occurring on the same side of the diaphragm. Stage 1E involves one organ or site outside the lymph system, while Stage 2E involves one extranodal site plus nearby lymph nodes, or multiple node groups, all on one side of the diaphragm. Based on these criteria, the patient was staged as Stage 2 primary extranodal lymphoma (2E). The staging bone marrow biopsy showed no evidence of lymphoma involvement. The patient was treated with rituximab, cyclophosphamide, doxorubicin, vincristine, and prednisone (R-CHOP) with significant improvement.

## Materials and methods

Hematoxylin and eosin (HE) and IHC stains were performed on formalin-fixed, paraffin-embedded tissue sections at our institution.

The systemic literature search was performed via the PubMed database with specific keywords: “primary laryngeal lymphoma.” This search identified approximately 165 case reports. From these 165, 56 cases of primary laryngeal B-cell lymphoma were identified, with some reports describing multiple cases (Table 1).

**Table 1:** Summary of reported cases of primary laryngeal B-cell lymphoma identified through systematic literature review

#	Histology Subgroup	No	Age (Range)	Sex (M/F)	Predominant Symptoms	Disease Stage (I/II)	Dominant Treatment	IHC Profile	Outcome	Ref
1	**Diffuse large B-cell lymphoma**	23	57.3 (13–85)	17/6	Hoarseness, dysphagia, dyspnea, B symptoms	74%	Chemotherapy-based (61%)	CD20 +; CD3 −; BCL6/BCL2/CD10/ MUM1 variable	Mostly no recurrence (6 m–6 y)	[20–37]
2	**MALT lymphoma**	19	62.1 (27–88)	9/10	Hoarseness/ dysphonia, dyspnea/stridor, dysphagia	63%	Radiotherapy-based (47%)	CD20 +; CD3 −/CD5 −/CD10 −/Cyclin D1 −; BCL2/PAX5/CD79a variable	Mostly no recurrence (4 m–4 y)	[38–54]
3	**Mantle cell lymphoma**	6	66.0 (43–84)	3/3	Dyspnea, hoarseness/ dysphonia, sore throat	50%	Chemotherapy-based (50%)	CD20 +; Cyclin D1 +/limited reported	Mostly no recurrence (5 m–7 y)	[55–60]
4	**B-cell lymphoma, Not Specified**	4	26.0 (4–76)	2/2	Hoarseness; dysphagia; weight loss	25%	Chemotherapy-based (100%)	CD20 +; CD45/EBER/MUM1/CD138/CD30 variable	Mostly no recurrence (5 m–3 y)	[61–64]
5	**Burkitt lymphoma**	2	51.5 (13–90)	1/1	Hoarseness, Neck Mass	50%	Chemotherapy-based (50%)	CD20 +; cMYC +; CD10/BCL6/PAX5/EBER variable; BCL2 − (reported in 1 case)	1 N/A; 1 death at 3 months (aspiration pneumonia)	[65, 66]
6	**Composite lymphoma**	2	43.0 (43)	2/0	Dysphagia, Neck Mass, weight loss	N/A	Chemotherapy-based (100%)	DLBCL component: CD20 +, BCL6 +, CD10/BCL2 variable; TdT −/CD3 −	Death n = 1; outcome N/A n = 1	[67, 68]
7	**Small lymphocytic lymphoma**	2	72 0.0 (67–77)	1/1	Hoarseness, (± foreign body sensation)	100%	Surgery + Radiotherapy-based	Limited reporting; CD20 +/CD45 +	Death (n = 2); 36 m and 162 m	[36, 57]
8	**Follicular** **lymphoma**	2	66.5 (62–71)	0/2	Hoarseness, Foreign body sensation	100%	Radiotherapy-based	CD20 +; CD45 +	Death (n = 2); 67–228 m	[36]
9	**Lymphoplasmacytic lymphoma**	1	72.0 (72)	1/0	Dysphagia	N/A	Surgery	Pos: CD20, CD138, CD19, IgG Neg: IgA, IgM, CD56	No recurrence (24 m)	[67]
10	**High-grade B-cell lymphoma**	1	41.0 (41)	1/0	Dyspnea, Hoarseness	N/A	Radiotherapy-based	Pos: CD20, L26 Neg: CD3	**Death** **(13 m)**	[68]

## Discussion

Classic FL of the larynx is an exceedingly rare diagnosis, previously reported only once in 1997 by Dr. Ansell and colleagues in their study of primary laryngeal lymphomas [11]. Another large study conducted by Aceñero et al. reviewed 2631 laryngeal biopsy samples over a period of 10 years and found only one case of lymphoma, a diffuse large B-cell lymphoma (DLBCL). Of the 737 carcinomas, 726 represented squamous cell carcinomas. The remaining cases included chondrosarcoma, liposarcoma, synovial sarcoma, neurinoma, and plasmacytoma [12]. These results suggest that lymphomas likely constitute fewer than 1% of laryngeal neoplasms.

Although primary laryngeal lymphoma is such a rare diagnosis, imaging findings can suggest an alternate diagnosis to the much more common squamous cell carcinoma. Multiple reviews of imaging results in such cases indicate that lymphomas in the larynx commonly show homogeneous enhancement on CT or T1-weighted MRI images; in contrast, squamous cell carcinomas will typically show signs of central necrosis [13–15]. Additionally, laryngeal lymphoma nearly always involves the supraglottic region, as was seen in our case [13–16]. This is likely due to the increased mucosal and lymphoid tissue in this region. In cases where a mass is identified superior to the glottis, a diagnosis of lymphoma should certainly be considered.

According to the 5th edition of the World Health Organization (WHO) Classification of Hematolymphoid Tumors (WHO HAEM5) criteria, follicular lymphoma is a neoplasm of germinal-center (GC) B-cells with varying proportions of centrocytes and centroblasts or large transformed cells and at least a partially follicular growth pattern [6, 17]. As B-cell tumors, they express the pan B-cell antigens such as CD19, CD20, and PAX5. Additionally, since they are derived from germinal centers, they express associated markers such as CD10 and BCL6 [6, 18]. As per the new WHO classification, there is no conclusive clinical evidence to justify distinguishing FL grade 1, grade 2, and grade 3 A as separate categories, although the International Consensus Classification (ICC) classification continues to use previously defined grading standards [18, 19].

We performed a systemic literature search via PubMed database with the keywords: “primary laryngeal lymphoma.” This search yielded 166 case reports; among reports available in the English language, 62 cases were identified describing primary laryngeal B-cell lymphomas. These reports described several types of lymphoma, such as Burkitt lymphoma (BL), DLBCL, MALT lymphoma, mantle cell lymphoma (MCL), and marginal zone lymphoma (MZL). Table 1 is made to present patients’ initial complaints, staging, immunohistochemical findings, treatment, and survival in an organized fashion. There is only one manuscript describing two cases of FL at the same time [11]. The median age from this review was 62 years old. There were 37 male and 25 female cases, and a wide variety of immunohistochemical stains were used for diagnosis.

## Conclusion

In conclusion, we report an unusual case of FL presenting as a laryngeal mass. Furthermore, we conducted an extensive literature review and discussed the relevant imaging and histopathological findings crucial to the diagnosis of primary laryngeal lymphoma.
